# Mechanical Properties and Decomposition Behavior of Compression Moldable Poly(Malic Acid)/*α*-Tricalcium Phosphate Hybrid Materials

**DOI:** 10.3390/polym17020147

**Published:** 2025-01-09

**Authors:** Shuta Hara, Akiko Kojima, Atsushi Furukawa, Takeshi Toyama, Hiroki Ikake, Shigeru Shimizu, Kimio Kurita

**Affiliations:** 1 Department of Material and Life Chemistry, Kanagawa University, 3-6-1, Kanagawa-ku, Yokohama 221-8686, Japan; ft102160vg@kanagawa-u.ac.jp; 2 Department of Materials and Applied Chemistry, College of Science and Technology, Nihon University, 1-8-14 Kandasurugadai, Chiyoda-ku, Tokyo 101-8308, Japantouyama.takeshi@nihon-u.ac.jp (T.T.); shimizu.shigeru@nihon-u.ac.jp (S.S.);

**Keywords:** Poly(malic acid), α-Tricalcium phosphate, Hybrid materials, The bone tissue material

## Abstract

Calcified tissues in living organisms, such as bone, dentin, and enamel, often require surgical intervention for treatment. However, advances in regenerative medicine have increased the demand for materials to assist in regenerating these tissues. Among the various forms of calcium phosphate (CaP), tricalcium phosphate (TCP)—particularly its α-TCP form—stands out due to its high solubility and efficient calcium release, making it a promising candidate for bone regeneration applications. Nevertheless, its rapid dissolution rate presents challenges when used as a reinforcing agent. In this study, we developed a hybrid material composed of poly(malic acid) (PMA) and α-TCP to achieve controlled calcium release while maintaining mechanical strength. The hybrid materials were prepared using a compression molding method optimized to suppress the hydrolysis of PMA. The bond between the carboxyl group of PMA and α-TCP was confirmed through infrared (IR) spectroscopy. A calcium release test demonstrated that the interaction between PMA and α-TCP extends the dissolution period of both components. These findings indicate that PMA/α-TCP hybrid materials have significant potential for applications in bone tissue engineering.

## 1. Introduction

The calcified tissues in living organisms are classified into three primary components, bone, dentin, and enamel, all of which often require surgical intervention for treatment [1,2,3,4]. However, with recent advances in regenerative medicine, there is an increasing demand for the development of materials that can assist in the regeneration of these tissues [5,6,7]. A key requirement for such regeneration-assisting materials is not only the mechanical reinforcement of damaged calcified tissues but also the supply of essential ions necessary for tissue recovery. Calcium phosphate (CaP) has been extensively studied as a material that aligns with these requirements [8,9]. In particular, the ability to control calcium release is crucial, and factors such as the size of CaP particles—ranging from microparticles and nanoparticles to amorphous nanodroplets—play a significant role. However, CaP exists in various crystalline forms, and it is essential to select the appropriate crystal form for controlled calcium release.

CaP in particulate form includes hydroxyapatite (HAP), tricalcium phosphate (TCP), and amorphous calcium phosphate (ACP) [10,11,12]. Among these, TCP is noted for its high calcium release behavior [12,13]. TCP exists in two primary crystalline forms, monoclinic α-TCP and rhombohedral β-TCP, with α-TCP offering better solubility than β-TCP [14,15]. Due to its high solubility, α-TCP is particularly effective in bone treatments, serving as a carrier for delivering growth factors that promote remineralization, as well as drugs such as simvastatin [16]. However, when α-TCP is used as a reinforcing agent for calcified tissues, its rapid dissolution rate presents a challenge [17]. Therefore, it is necessary to develop a binder that can both enhance the mechanical strength of α-TCP microparticles and regulate calcium release over a controlled timeframe.

Hybrid materials combining polymers with inorganic microparticles have been extensively studied for their excellent bioactivity and ability to support cellular functions such as adhesion, migration, and proliferation in osteoblasts and fibroblasts [8,18,19,20]. A critical factor in the development of these materials is the design of the interaction between the inorganic particles and the polymer matrix. Proper interaction not only enhances the overall mechanical strength of the material but also prevents phase separation between the polymer and inorganic particles [21,22,23]. Silk fibroin has been widely researched due to its high biocompatibility and the presence of functional groups like carboxyl (COOH), carbonyl (CO), and hydroxyl (OH), which facilitate binding with α-TCP. However, silk fibroin is difficult to melt, potentially causing issues with moldability in dry environments [24,25,26]. In contrast, biocompatible polyesters such as polylactic acid are easily moldable through heating.

Among biocompatible polyesters, Poly(malic acid) (PMA) has garnered attention as a promising material for a wide range of biomedical applications, such as drug delivery and tissue engineering [27,28]. PMA is unique in that it is the only naturally occurring water-soluble polyester, characterized by its abundant carboxylate groups [29,30]. PMA undergoes complete biodegradation into L-malic acid upon hydrolysis, ensuring excellent biocompatibility while minimizing immunogenicity and cytotoxicity [31]. Additionally, PMA’s rapid decomposition rate is advantageous, as malic acid is naturally metabolized into oxaloacetic acid via the tricarboxylic acid cycle [32]. Due to these properties, PMA has recently been reported to be highly useful as a carrier for poorly soluble small-molecule anticancer drugs [33]. Given its non-toxic and non-immunogenic nature, along with its high density of carboxyl groups, PMA is considered a promising candidate for calcium delivery systems. However, to date, no studies have reported the use of PMA in this specific application.

**Figure 1:**
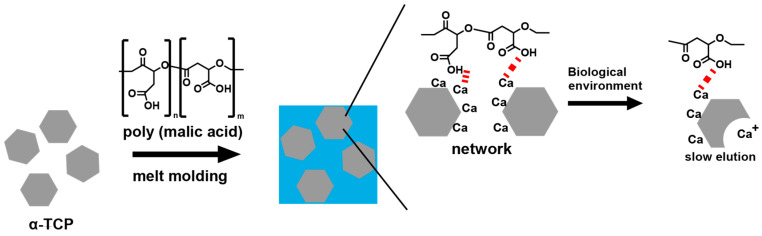
Illustration of the preparation method of a-TCP and PMA hybrid material and its dissolution resistance mechanism.

In this study, by leveraging the interaction between the carboxyl groups of PMA and α-TCP, we demonstrate that the hybrid material can be easily molded using a compression molding technique while achieving controlled dissolution of α-TCP (Figure 1). The PMA was a low molecular weight ((Mw) of 3100) compound chemically polymerized using a tin catalyst due to a focus on industrial-scale production. To optimize the mixing ratio of PMA and α-TCP, we adjusted the molding temperature, molding time, and proportions of PMA and α-TCP. We then performed bending and compression tests on the resulting samples. Furthermore, the bonding interactions between the carboxyl groups of PMA and α-TCP were analyzed using infrared (IR) spectroscopy. The morphology of the samples was observed using scanning electron microscopy (SEM). Finally, dissolution tests of α-TCP in a simulated biological environment were conducted to evaluate the effectiveness of these hybrid complexes.

## 2. Materials and Methods

### 2.1. Materials

L-malic acid, deuterium oxide, and tin(II) chloride were purchased from Sigma-Aldrich (St. Louis, MO, USA). Tetrahydrofuran, α-cyano-4-hydroxycinnamic acid, and phosphate buffer solutions (pH = 7.4) were obtained from Kanto Chemical (Chuo-ku, Tokyo, Japan). α-Tricalcium phosphate (α-TCP) with a particle diameter of approximately 64 μm was also purchased from Kanto Chemical (Chuo-ku, Tokyo, Japan).

### 2.2. Synthesis of Poly(Malic Acid) (PMA)

The PMA was synthesized using a direct polymerization method based on previously reported procedures [34,35]. To begin, 13.4 g of L-malic acid was placed in a stoppered round-bottomed flask and heated to 102 °C until the L-malic acid melted. Then, 0.38 wt% tin(II) chloride was added to the melted L-malic acid. The pressure was reduced to 1 mmHg under a nitrogen atmosphere, and the mixture was heated to 110 °C until the tin(II) chloride completely dissolved. The polymerization reaction was subsequently carried out for 50 h with continuous stirring at 50 rpm. The α/*β* isomeric ratio of the product was determined using ^1^H NMR (Figure S1), showing a molar ratio of 6:4. The molecular weight (Mw) of the synthesized PMA was determined by MALDI-TOF-MS from Applied Biosystems (Warrington, Cheshire, UK), confirming an Mw of 3100.

### 2.3. Preparation of 4.5 μm α-TCP

The α-TCP with a particle size of 4.5 μm was prepared by crushing and sieving the original 64 μm α-TCP. These particle sizes were calculated according to Equation (1) by determining the specific surface area of the particles using the BET specific surface area testing device (Table S1).

(1) $$d_{p}=\frac{6}{S_{W}\cdot \rho }$$

where $d_{p}$ is the particle diameter [µm], $S_{W}$ is the specific surface area [m^2^/g], and ρ is the density of α-TCP [g/cm^3^].

### 2.4. Preparation of α-TCP/PMA Hybrid Materials

The α-TCP and PMA were kneaded at 0 °C for 2 h, and mixed in the desired mixing ratio of α-TCP/PMA, such as 35/65, 50/50, and 65/35 by weight fraction. The resulting mixture was placed in a mold, and compression molding was performed under a pressure of 10 MPa at temperatures of 110 °C, 130 °C, and 150 °C. The samples were compression molding and obtained 10 mm × 20 mm × 5 mm pieces. After molding, the samples were vacuum-dried for one day before further analysis.

### 2.5. Characterization

The BET specific surface area of the α-TCP particles was determined using a specific surface area testing device (Gemini 2360, Shimadzu Corporation, Kyoto, Japan). The mixing ratio of α and β types in PMA was determined using ^1^H NMR spectroscopy (JNM-ECX400, JEOL Ltd., Tokyo, Japan). A scanning electron microscope (S-3000N, Hitachi High-Tech Corp., Tokyo, Japan) was used to observe the morphology of the hybrid material composed of α-TCP and PMA with different particle sizes. The compressive strength of the hybrid materials was tested using a Universal Strength Testing Machine (CT-1000, Marubishi Scientific Instrument MFG Co. Ltd., Tokyo, Japan). Bending strength tests were conducted using a Precision Universal Tester (PL-300, Marubishi Scientific Instrument MFG Co. Ltd., Tokyo, Japan). Infrared absorption spectra were measured using the KBr method on a VALOR-III spectrometer (JASCO Corp., Tokyo, Japan). Thermogravimetric analysis (TGA) was performed with a Thermo Plus TG8120 (RIGAKU Corp., Tokyo, Japan) in an air atmosphere, with a temperature ramp of 5 °C/min. Hydrolysis tests of the hybrid materials were carried out using an Accumet Model-15 pH meter (Thermo Fisher Scientific K.K., Tokyo, Japan).

## 3. Results and Discussion

### 3.1. Preparation of PMA/α-TCP Hybrid Materials

**Figure 2:**
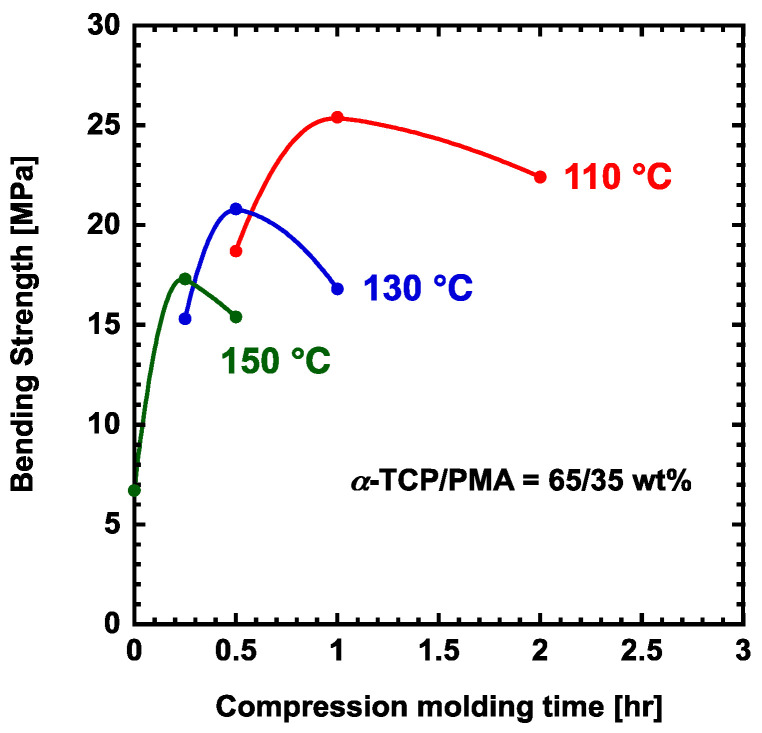
Relationship between flexural strength and compression molding temperature of α-TCP/PMA hybrid materials prepared from α-TCP.

To investigate the influence of compression molding conditions on the bending strength of the composite, a series of tests were conducted by varying the molding parameters, specifically the molding time and temperature, using a mixture of α-TCP and PMA at a 65/35 wt% ratio (Figure 2). The glass transition temperature of PMA has been reported to be approximately 1 °C, however, it has been suggested that PMA may form hydrogen bonds [36]. Therefore, considering the flowability of PMA during molding, the lower limit temperature was set to 110 °C. The maximum bending strength observed at different temperatures showed a decreasing trend in the following order: 110 °C (25 MPa), 130 °C (21 MPa), and 150 °C (17 MPa). Furthermore, the time required to achieve the maximum bending strength at each temperature also decreased, with the order being 110 °C (1 h), 130 °C (0.5 h), and 150 °C (0.25 h). These results suggest that the decomposition of the PMA during the compression molding process is due to the basic interface with the α-TCP, which could promote the hydrolysis of the PMA. Based on these results, the optimal molding conditions for the next experiments were established as 110 °C for a duration of 1 h.

### 3.2. Characterization of PMA/α-TCP Hybrid Materials

**Figure 3:**
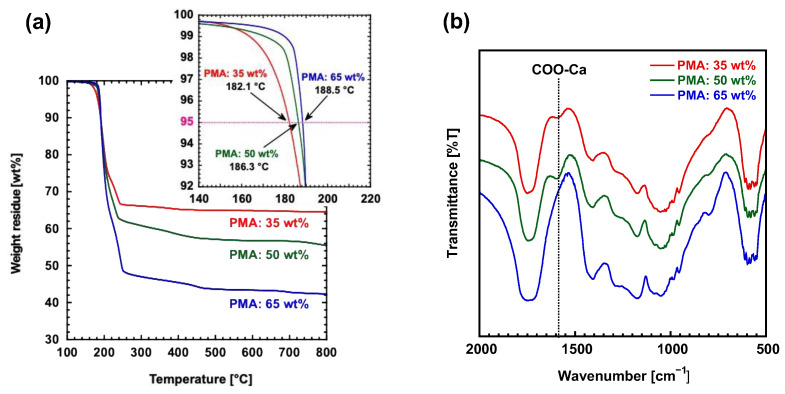
(**a**) TGA curves for each kind of hybrid material molded at 110 °C. The TGA curves of the upper frame mean the 5 wt% weight loss of each hybrid material, i.e., the thermal decomposition temperature. (**b**) IR of all hybrid materials is performed to confirm the binding of calcium to the carboxy groups of PMA.

In order to thoroughly investigate the effect of the blend ratio of α-TCP and PMA on the binding of carboxyl groups to calcium, as well as its subsequent impact on the morphology of the hybrid materials, a series of samples were prepared with different blend ratios of PMA, specifically at 36 wt%, 50 wt%, and 65 wt%. Thermogravimetric analysis (TGA) was conducted on each hybrid material to accurately determine the actual mixing ratios achieved (Figure 3a). The results of the TGA confirmed that in all the samples, the actual weight ratio of the added PMA was lower than the originally charged amount. Furthermore, it was observed that as the amount of PMA increased, the rate of weight reduction also increased. This phenomenon is believed to be caused by the progression of intramolecular dehydration that occurs when PMA is subjected to heat during the molding process. The temperatures for 5 wt% weight loss of PMA are as follows: 35 wt% (182.1 °C), 50 wt% (186.3 °C), and 65 wt% (188.5 °C). As the amount of α-TCP increases, the temperature decreases. These results support the possibility that α-TCP is involved in the degradation of PMA. Figure S2 shows the IR spectrum of PMA. A sharp peak at 1730 cm^−1^ indicates C=O stretching, a moderate peak at 1450 cm^−1^ corresponds to O-H bending of carboxylic acid, multiple peaks at 1200–1100 cm^−1^ are due to C-O stretching, and weak peaks at 950–800 cm^−1^ are attributed to out-of-plane C-H bending in the methylene groups within the PMA main chain. The infrared (IR) spectrum of each hybrid material is presented in Figure 3b. In the hybrid samples containing 35 wt% and 50 wt% PMA, a distinct peak at 1580 cm^−1^, corresponding to the COO-Ca bond, which indicates the interaction between the calcium and carboxyl groups, was observed. However, in the sample with 65 wt% PMA, this peak was notably absent. In the hybrid samples with 35 and 50 wt% PMA, a peak of 1580 cm^−1^ (COO-Ca) related to the interaction between the calcium and carboxyl groups was observed, but this was not seen in the 65 wt% PMA sample.

**Figure 4:**
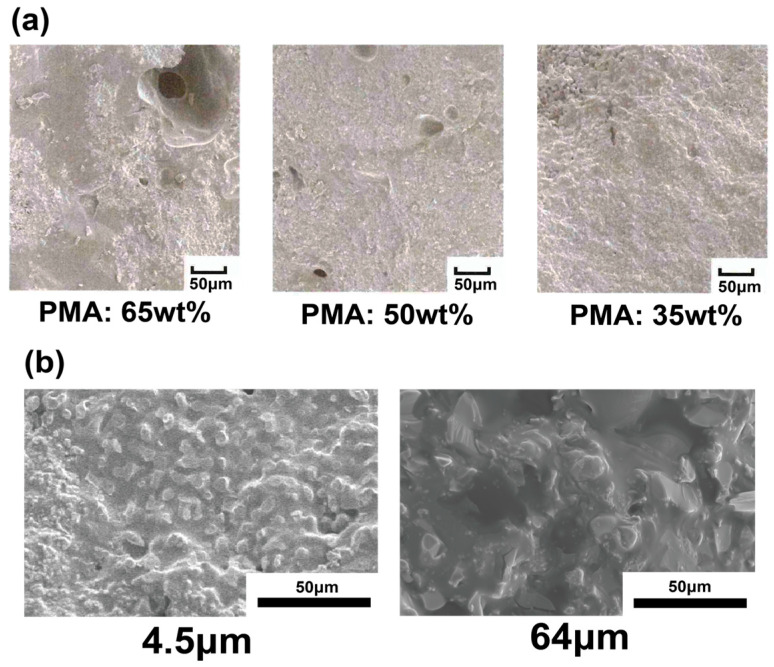
(**a**) SEM images of hybrid materials prepared with different ratios of α-TCP/PMA hybrid materials. (**b**) SEM images of α-TCP/PMA (containing 35 wt%) hybrid materials prepared from α-TCP with a particle size of 4.5 μm and 64 μm. The black bars in the figure indicate the 50 μm scale.

Figure 4a displays scanning electron microscopy (SEM) images of the morphology of each hybrid material. A clear tendency was noted, in which the number of voids present in the material increased as the weight ratio of PMA increased. This can be attributed to the fact that as the amount of PMA increases, the volume of the PMA domain within the material also increases. Consequently, the proportion of PMA that is able to effectively bind to the α-TCP decreases. This reduction in binding weakens the interactions between the α-TCP particles, thereby making the formation of voids more likely. These observations are consistent with the data obtained from the IR analysis. To further explore the influence of PMA domains on the particle size of α-TCP, an additional hybrid material containing 35 wt% PMA was prepared using α-TCP with a larger particle size of 64 μm and was compared to the sample that used α-TCP with a particle size of 4.5 μm (Figure 4b). It was found that the hybrid material with 64 μm α-TCP exhibited larger PMA domains when compared to the sample prepared with 4.5 μm α-TCP particles.

### 3.3. Properties of Hybrid Materials

**Figure 5:**
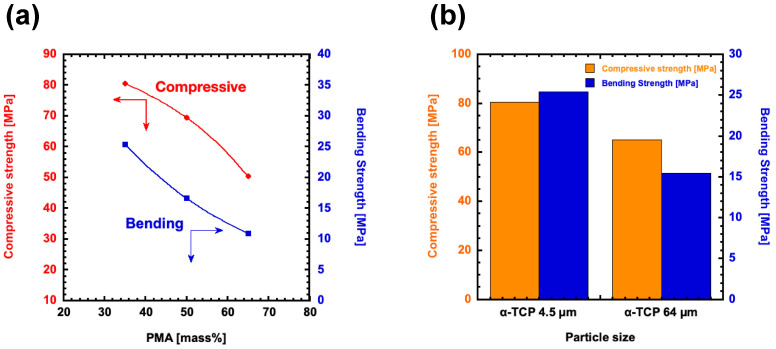
(**a**) Bending strength and compressive strength of hybrid materials prepared with different PMA mixing ratios. (**b**) A comparison of the bending strength and compressive strength made for 35 wt% PMA hybrid materials prepared with different sizes of α-TCP.

Figure 5a presents the results of mechanical tests conducted under various molding conditions. The data clearly show that both the bending strength and compressive strength of the materials increased as the mixing ratio of PMA decreased. When comparing the rate of change between the bending strength and compressive strength, it was observed that the compressive strength tended to decrease at a faster rate than the bending strength as the PMA ratio increased. This trend suggests an increased presence of voids, as observed in the SEM images, which becomes more prominent with higher PMA content. These voids are likely disrupt the material’s ability to withstand compressive loads, resulting in a more rapid reduction in compressive strength compared to bending strength. Furthermore, the microstructural changes induced by the higher PMA content may influence the stress distribution within the material, further contributing to the observed decrease in compressive strength. Additionally, a hybrid material containing 35 wt% PMA with α-TCP was carried out to measure both the bending strength and compressive strength. The results demonstrated that, when compared to the hybrid material prepared with α-TCP particles of 4.5 μm, the sample with smaller particle size exhibited bending strength that was 1.6 times higher and compressive strength that was 1.3 times higher, as shown in Figure 5b. In the hybrid material with a PMA ratio of 35 wt%, no voids were detected regardless of the α-TCP particle size used (Figure 4b). These findings highlight the significant impact of α-TCP particle size on the mechanical properties and microstructural features of the hybrid material. The smaller α-TCP particle size (4.5 μm) led to higher bending and compressive strength than the larger particle size (65 μm), likely due to a reduction in PMA domain size. Furthermore, the presence of the COO-Ca bond, as indicated by the peak at 1580 cm^−1^ in both samples, suggests that the chemical interaction between the α-TCP and PMA components is consistent across different α-TCP particle sizes. Therefore, it is considered important to adjust the α-TCP particle size and the PMA content in order to control the PMA domain size and, consequently, the mechanical strength.

**Figure 6:**
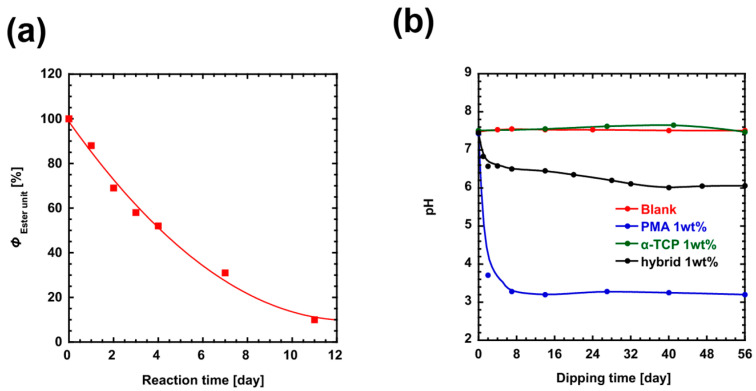
(**a**) Correlation between the *Φ*_Ester unit_ values of ester units remaining in PMA in hydrolysis reactions carried out in phosphate buffer (pH = 7.4) at 37 °C in a warm bath and the reaction time. (**b**) Correlation between immersion time and pH of 1 wt% of PMA, α-TCP (particle size: 4.5 µm), and α-TCP/PMA hybrids (containing 35 wt%, molding conditions 110 °C, 1 h) in phosphate buffer solution (pH = 7.4). (**b**) Correlation between immersion time and pH of 1 wt% of PMA, α-TCP (particle size: 4.5 µm), and α-TCP/PMA hybrids (containing 35 wt%, molding conditions 110 °C, 1 h) in phosphate buffer solution (pH = 7.4).

Solubility tests were conducted using the hybrid material containing 35 wt% PMA, as this composition demonstrated the highest bending and compressive strength in previous mechanical tests. First, the hydrolysis process of PMA was evaluated under conditions mimicking the biological environment. The PMA was incubated at 37 °C in a phosphate buffer solution with a pH of 7.4, and ^1^H NMR spectroscopy was used to monitor its degradation over time. The phosphate buffer solution was prepared with heavy water to maintain a weight ratio of 1 wt% between the phosphate buffer solution and the PMA. During the hydrolysis process, the proton signal of the alkoxy group in the PMA main chain shifted from 5.5 ppm to 4.45 ppm, indicating that hydrolysis was occurring. The PMA structure included ester units, which were defined in Equations (2) and (3) based on their integral ratios. The relative number of ester groups: $\Phi_{Ester\;unit}$ was plotted as a function of the number of incubation days (Figure 6a).

(2) $$\Phi_{Ester\;unit}\equiv \frac{\left[5.5\;ppm\;ratio\right]}{\left[5.5\;ppm\;ratio\right]+\left[4.45\;ppm\;ratio\right]}\times 100$$

(3) $$\Phi_{Ester\;unit}=\frac{\left[Ester\;ratio\right]}{\left[Total\;malic\;acid\;unit\right]}\;\%$$

As a result of this analysis, it was confirmed that 50% of the PMA had undergone hydrolysis within 3 days, and approximately 90% of the PMA was hydrolyzed after 11 days of incubation. To further investigate the decomposition behavior, 1 wt% of pure PMA, α-TCP, and the hybrid material were each added to separate phosphate buffer solutions, and the decomposition process of each material was evaluated by measuring changes in the pH of the solution (Figure 6b). For the pure PMA sample, the pH dropped rapidly in the initial phase and then stabilized after 11 days. Based on the results of the NMR analysis, this rapid decrease in pH can be attributed to the generation of carboxyl groups that occur as a byproduct of PMA hydrolysis. In contrast, the hybrid material exhibited a different pattern of pH change. The pH of the solution initially decreased rapidly within the first 3 days, but after this point, the rate of pH change slowed, with the pH gradually changing over a period of 32 days. For the α-TCP sample, however, there was no significant change in pH, even after 56 days of incubation. This stable pH behavior is likely due to the common ion effect, where the phosphate ions released from the dissolving α-TCP interact with the phosphate buffer, preventing large pH shifts. Therefore, the decomposition rate of the hybrid material can be assessed by tracking the pH changes attributed to the release of carboxyl groups from the PMA component. It was observed that the dissolution rate of the hybrid material was slower than that of pure PMA, indicating a delay in its decomposition. This deceleration is thought to be due to the effective binding between α-TCP and PMA via the COO-Ca bond, which provides stability to the hybrid material. Based on the results of these experiments, it is estimated that the hybrid material will fully dissolve over the course of approximately one month. These findings suggest that the synergistic interaction between PMA and α-TCP allows for precise control over the time required for the hybrid material to decompose into malate ions and calcium ions in vivo. This controlled degradation behavior could have significant implications for the design of biomaterials with tunable degradation rates in biological environments.

## 4. Conclusions

Hybrid materials composed of PMA and α-TCP were developed to address the rapid dissolution challenges of α-TCP in biological environments. By optimizing the blend ratios and molding conditions, it was confirmed that the material could be molded while preserving the COO-Ca bond. Mechanical tests demonstrated that both bending and compressive strengths increased as PMA content decreased. Moreover, using 4.5 μm of α-TCP improved the material’s mechanical strength by reducing PMA domain size. Solubility tests under simulated in vivo conditions indicated that calcium ion release from α-TCP could be controlled over a period of one month. These findings suggest that the hybrid materials achieve both enhanced mechanical strength and functionality as calcium delivery carriers. In the future, further reducing α-TCP particle size could decrease the PMA domain size and increase the COO-Ca bonds, thereby prolonging calcium retention in the body. This synergistic combination of PMA and α-TCP can be tailored to meet specific needs in regenerative medicine, offering a promising approach for designing biodegradable biomaterials with adjustable degradation rates. The results provide a foundation for future research to further optimize these hybrid materials for various tissue engineering applications.

## Data Availability

No new data were created or analyzed in this study. Data sharing is not applicable to this article.

## Supplementary Materials

The following supporting information can be downloaded at: https://www.mdpi.com/article/10.3390/polym17020147/s1, Figure S1: ^1^H NMR spectrum of PMA composed of α- and *β*-type at a molar ratio 6:4; Figure S2: IR of PMA; Figure S3: FT-IR spectrum of a PMA/α-TCP hybrid material comprising PMA weight fraction: 35 wt% and α-TCP particle size: 64 μm; Table S1: Grain size of α-TCP as raw material and α-TCP grain size after grinding and sieving the raw material.
