# Supplementary material for: Mechanical Properties and Decomposition Behavior of Compression Moldable Poly(Malic Acid)/α-Tricalcium Phosphate Hybrid Materials
Source: Polymers (Basel). 2025 Jan 9;17(2):147. doi: 10.3390/polym17020147 (PMC11768388; doi:10.3390/polym17020147)
Supplement: Supplementary file 1 [file polymers-17-00147-s001.zip › polymers-3264329-supplementary.pdf]

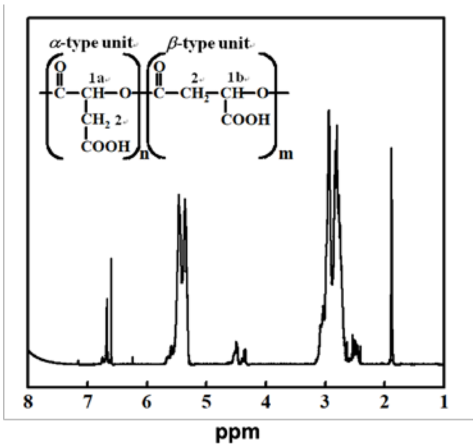

Figure S1. ^{1}H NMR spectrum of PMA composed of α- and β-type at a molar ratio 6:4.

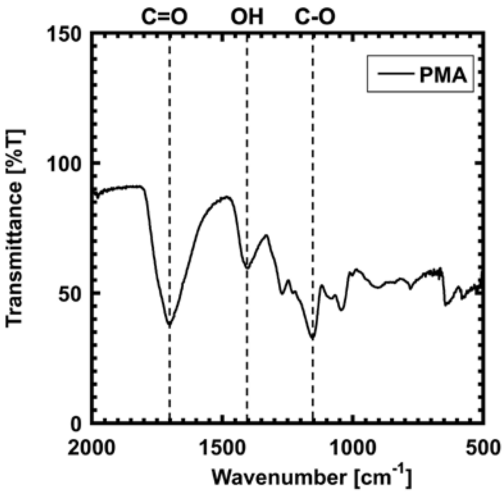

Figure S2. IR of PMA

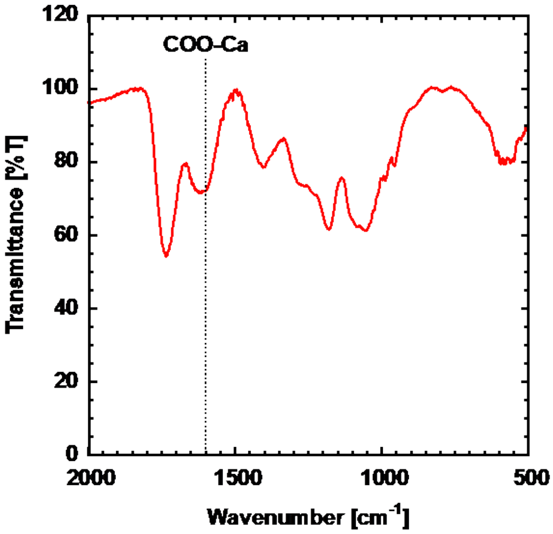

Figure S3. FT-IR spectrum of a PMA/α-TCP hybrid material comprising PMA weight fraction: 35 wt% and α-TCP particle size: 64 μm.

Table S1. Grain size of α-TCP as raw material and α-TCP grain size after grinding and sieving the raw material.

	Surface Area* [m/g]	Density [g/cm^3]	Grain Size [μm]
α-TCP	0.033	2.86	63.8
	0.464	2.86	4.52

*Determined by BET
